# Supplementary material for: The Construction and Exploration of a Comprehensive MicroRNA Centered Regulatory Network in Foxtail Millet (Setaria italica L.)
Source: Front Plant Sci. 2022 May 6;13:848474. doi: 10.3389/fpls.2022.848474 (PMC9121102; doi:10.3389/fpls.2022.848474)
Supplement: Supplementary file 2 [file Data_Sheet_2.zip › Sit-miR2275b_Seita.3G014700.1_1136_TPlot.pdf]

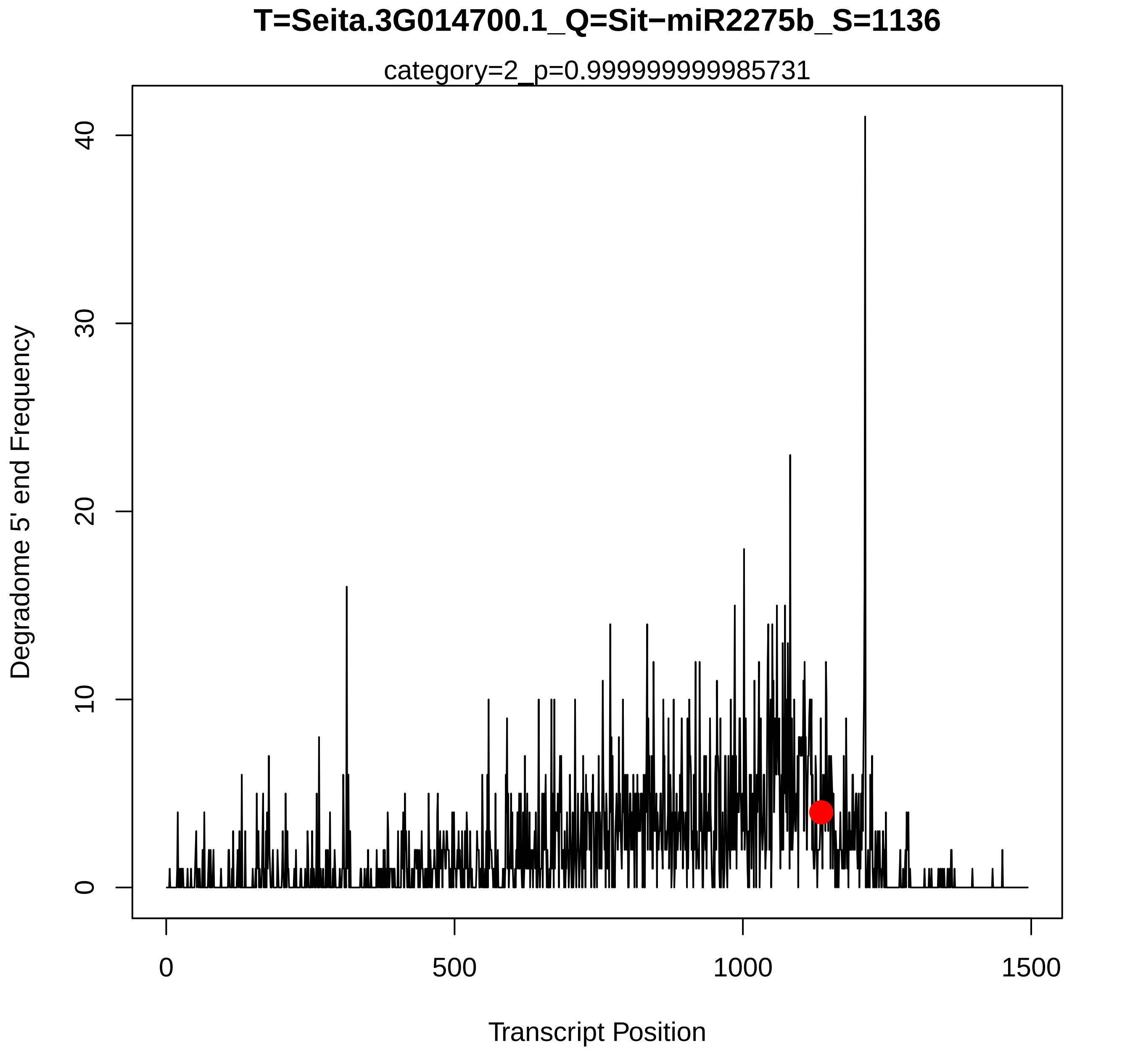
T=Seita.3G014700.1_Q=Sit−miR2275b_S=1136
category=2_p=0.999999999985731
Degradome 5' end Frequency
0
10
20
30
40
0
500
1000
1500
Transcript Position
